# Book Reviews

**Published:** 1800-06

**Authors:** 


*Annals of Medicine for the Year* 1799; *exhibiting a conciſe View of the lateſt and moſt important Diſcoveries in Medicine and Medical Philoſophy.* By A. DUNCAN, Sen. M. D. and A. DUNCAN, Jun. M. D. &c.

[ Continued from pp. 482—486 of our laſt. ]

V. *Obſervations on the Pemphigus Major of Sauvages.* By Dr. R. HALL, Phyſician, Jedburgh.

In a former paper Dr. Hall had given it as his opinion, that Pemphigus is merely a ſporadic diſeaſe. Some other practitioners believe it to be contagious. "An opportunity ſoon occurred of ſubmitting this opinion to the teſt of actual experiment, by the re-appearance of the diſeaſe, in one of the two patients who had been ſubjected to this affection, nearly about the ſame period, in the preceding year.

"Mrs. H. had, for a few weeks previous to the preſent attack, been occaſionally ſubject to ſlight febrile paroxyſms, for which bark, &c. had been preſcribed, but were never diligently employed.

"Towards the evening of the 28th July, ſhe was ſeized with giddineſs and head-ach, a ſenſe of great laſſitude and weakneſs, with other precurſory ſymptoms of fever. On the following morning, her ſkin was preternaturally hot; pulſe frequent, weak, and irregular; head-ach rather more violent; reſpiration ſomewhat oppreſſed; was thirſty, but not coſtive; her tongue parched, but not foul. She had paſſed a reſtleſs and uneaſy night, and ſaid, that ſhe now apprehended the nature of her diſeaſe would prove ſimilar to that which ſhe had experienced laſt year. In the evening, a ſingle veſicle appeared on the thigh.

"30th. She had been equally reſtleſs as on the foregoing night; and to an aggravation of the former ſymptoms were now ſuperadded, great irritability of the ſyſtem, and frequent, but irregular ſhiverings. In the courſe of the day, five more veſicles made their appearance on different parts of the body.

"Auguſt 1. A ſmall one appeared on the exterior part of the meatus auditorius. By the evening of the next day, all the febrile ſymptoms had ſuffered a conſiderable abatement; but ſhe continued, for a few days, affected with much languor and debility; had a ſlight exacerbation of fever every night, with an evident apyrexia in the morning.

"Towards

"Towards the decline of the complaint, an eruption of ſmall pimples came on, eſpecially about the neck and arms, ſimilar to thoſe excited by nettle burning; but which ſoon went off, without any bad ſymptom. The diſeaſe was mild, in compariſon with that ſhe had ſuſtained in the preceding year, and ſhorter in the term of its duration. The veſicles were few in number, and wholly confined to the external ſurface of the body. Thoſe that did appear, were filled, however, in like manner, with a yellowiſh ſerum, and of the ſame magnitude as on the former occaſion. They were painful upon being touched, but the circumjacent ſkin was not much inflamed. Of two or three that were punctured, ſhe complained a good deal, and obſerved, that her ſenſations after the operation were ſimilar, as ſhe conceived, to what would have been felt by her upon the application of any corroſive or cauſtic matter to the ſame part of the body. Upon the moſt diligent ſcrutiny, I could not diſcover that any perſon, either in the town or neighbourhood, had been affected with a ſimilar complaint; nor was the diſeaſe communicated to any one, although, both now and in the preceding attack, the patient had, at my particular requeſt, continued to allow a perſon to ſleep with her during the whole period of her illneſs.

"Both during the progreſs, and at the height of the diſeaſe, ſome of the fluid with which the veſicles were filled was taken, and with it I inoculated myſelf and two other perſons, in both arms, making three punctures in each arm. In one of the patients, on the day after the inſertion of the matter, a ſingle puncture exhibited a very ſlight degree of inflammation; not, however, more than what frequently occurs from a ſcratch or puncture made with a clean inſtrument: But neither in this patient, nor in the other two, was any conſtitutional effect, or the leaſt perceptible indiſpoſition, produced. The reſult of theſe attempts to communicate the diſeaſe, by inoculation and contact, although not perhaps ſufficiently numerous to prove deciſive of the queſtion, is at leaſt extremely unfavourable to the hypotheſis of thoſe who aſſert the contagious nature of pemphigus, and tends ſtrongly to ſupport and confirm the negative concluſion.

"From the foregoing ſtatement it would appear, that the following inferences may be fairly deduced.

"1ſt, That pemphigus is a diſeaſe of which perſons are ſuſceptible more than once in the courſe of their lives.

"2dly, That the diſeaſe originates where no ſource of infection can poſſibly be diſcovered, and ſeems generally connected with more or leſs of an affection of the whole ſyſtem.

"3dly, That patients labouring under it may have conſtant intercourſe with others, and yet never communicate the diſeaſe to any of them.

"4thly, That the diſeaſe is not communicable, like the cow-pox or ſmall-pox, by inoculation.

"On the whole, when we comprehenſively ſurvey the evidence recorded by recent writers on the ſubject, as well as that furniſhed by the preſent and former caſes, we muſt, I apprehend, be neceſ-

ſarily farily led to conclude, that the pemphigus major of Sauvages is an affection merely fporadic, and not of a contagious nature.

" That the fymptoms accompanying one or other inftances of this affection, are thofe which attend febrile difeafes, whether inflammatory or putrid, the cafes given by Drs. Dickfon and Stewart, and the one recorded by Mr. Chriftie, fufficiently evince. In practice, therefore, it would appear, the moft important diftinctions are, to afcertain,

" 1ft, When the fever is of an inflammatory nature, and accompanied with ftrong and increafed action of the vafcular fyftem.

" 2dly, When the fever has a tendency to the typhoid type; is marked by great debility, and fymptoms which denote a tendency of the fluids to putrefaction. In the firft cafe, it will be obvious, that evacuation and other antiphlogiftic remedies, fuited to the nature of the cafe, will be proper. As, on the other hand, in the fecond, it will be equally neceffary to fhun all evacuations, and to employ thofe remedies alone which fupport the ftrength, and give tone and vigour to the fyftem.

" From the whole concourfe of fymptoms, in the prefent, and in the two cafes of this affection formerly communicated, we are naturally led to infer, that the difeafe, in a great meafure, depended on a certain ftate of debility, and a tendency of the fluids to putrefaction.

" The general indications of cure thence deducible, are fufficiently obvious.

" In the cafe now under confideration, on the firft acceffion of the complaint, when the fkin was hot and dry, a mild antimonial was exhibited, in order principally to excite a gentle diaphorefis; but its ufe was foon difcontinued.

" Afterwards, opiates combined with vitriolic æther were found very ufeful in diminifhing the effects of irritation, and in promoting the determination to the furface. The bark and other tonics, particularly the nitrous acid, in a ftate of proper dilution, were early adminiftered, and proved very effectual in obviating the effects of debility. By thefe means, and the ulterior employment of other auxiliaries, the health of the patient was fpeedily re-eftablifhed."

VII. *Hiftory of a Cafe, terminating fuccefsfully, in which an inverted Uterus was extirpated.* By Mr. Alexander Hunter, Surgeon, Dumbarton.

We pafs over the firft part of this cafe, and only prefent our readers with the conclufion:

" When the womb firft came down, it was nearly of the fize of a fmall pine-apple, and felt hard. The fecond time it was fmaller, but ftill harder. Before returning it into the vagina, a trial was always made to reduce the inverfion of it; but after the firft time, the fundus was only dinted by any force that could be ufed.

" The profpect before the patient was now deplorable. The reftoring reftoring the uterus was abfolutely impracticable; and, if allowed to remain in its prefent fituation, it muft be very diftreffing.

"About a fortnight elapfed in this way, when a new fet of fymptoms took place. A difcharge of a thin watery nature began to flow from the whole furface of the womb, which gradually increafed in quantity, and became fo extremely fœtid, that it was very difagreeable to enter the room. And, though great attention was beftowed, the bed was always wet. Her ftrength was foon much reduced. And, notwithftanding a liberal ufe of bark, elix. vitriol. and port-wine, fevere hectic attacks came on.

"In this ftate of the bufinefs, no plan could be figured for faving the patient, without amputating the uterus. Every endeavour I had ufed for procuring information, either from medical men or books, left me ftill in the dark; as in every cafe of inverfion mentioned, not one was to be found, where the patient had furvived for any time, unlefs the womb was directly returned. But, after confidering that the organ was not immediately neceffary to life; that very extenfive wounds, even in its diftended ftate, has been made without any ill fymptoms; and that, in its prefent fituation, the functions were for ever deftroyed; indeed, that it was now only a burdenfome mafs; and the woman herfelf wifhing eagerly to be relieved from the miferable way fhe was then in, it was determined to extirpate it.

"I began the operation, by fixing a ftrong ligature on the neck of the tumor, clofe to the os externum. But being fearful of fpafmodic affections from this compreffion, I waited fix hours without proceeding farther. During all that time, however, no complaint was made, no pain was felt.

"With a fcalpel the whole uterus was then cut off, clofe to the ligature. Still neither fymptoms of pain, nor even uneafinefs, were perceived. And, I believe, the operation was over before the patient knew it had been begun. She was then laid to reft, and an opiate adminiftered.

"During the night fhe flept well; and, next morning, was very much refrefhed. The hectic fymptoms went off; her appetite returned; and, in fourteen days, fhe was able to get out of bed. At the end of a month fhe was perfectly recovered.

"Since that time fhe has enjoyed a very good ftate of health; except now and then fome touches of hyfteric head-ach, and fometimes ftitches and plethoric fymptoms in the fpring and fummer months. She does not menftruate, although ftill a young woman. She has a tendency to obefity, and even all her precautions cannot counteract it.

"From what happened in the preceding cafe, it will probably be allowed, that the womb, when not in an inflamed ftate, may be handled, or even wounded, without pain; that the whole of it may be cut off without injury; and that, in cafe of inverfion, attended with fevere flooding, if the womb cannot be returned, the hæmorrhage may be prevented, by tying a ligature round the neck of the uterus.

"Some

"Some months after this cafe happened, I gave the uterus to Dr. Jeffray, of Glafgow, who, I believe, ftill has it."

XII. *Obfervations on the Benefit derived from the Application of cold Water, in Cafes of Scarlatina Cynanchica.* By Dr. Geo. Mosman, Phyfician, Bradford.

"A boy, eight years of age, on the 31ft of July laft, was feized with great laffitude, with rigors, fucceeded by extreme heat, thirft, fore throat, and every fymptom characteriftic of that fpecies of pyrexia, denominated *Scarlatina Cynanchica*.

"On the following day, the apothecary to the family was confulted, and prefcribed an emetic, which operated well. He was then directed an aperient folution, which procured him feveral evacuations, without any abatement of fymptoms.

"On the morning of the 2d of Auguft, when I vifited him, the whole furface of his body was covered with a fcarlet eruption. His tongue was dry, and exhibited a fur approaching to black; the internal fauces were confiderably tumefied, and were of a deep red colour; his eyes had the appearance of an incipient fuffufion upon the tunica albuginea; his pulfe beat 135 ftrokes in the minute; his urine was fcanty, and fingularly pale.

"I had not an opportunity of applying to him the thermometer, but his fkin felt intenfely hot.

"Previous to the occurrence of this cafe, I had perufed Dr. Currie's admirable treatife on the ufe of cold water in fevers. As I have been in the habit for feveral years paft, of exhibiting and applying cold liquids in almoft every cafe of pyrexia, the practice was not new to me. But Dr. Currie's mode of application is different from mine. He directs the patient to be taken out of bed, during the hot ftage, and to have water thrown upon the whole furface of the body. I have conftantly recommended cold vinegar, or vinegar and water, to be applied, at the period fpecified, by means of a fponge.

The effects produced upon the fyftem by Dr. Currie's method and mine, are precifely fimilar; and I am confident, from an enlarged experience, that, if it be poffible to render the phenomena of fever lefs formidable, or to arreft their progrefs, the application of cold water is the inftrument to be employed.

"It is true, that the popular prejudice againft the expofure to cold in the hot ftage of fever, is remarkably ftrong, and I have found much difficulty in combating the error. When I once fucceed, however, in perfuading my patients to make the experiment, there needs little art to induce them to repeat it. The ufe of cold fluids is fo refrefhing to them, and produces fo complete a folution of the intenfe heat and reftleffnefs, under which they labour, that upon every recurrence of fimilar phenomena, they are eagerly folicitous for the fame grateful application.

The father of the boy whofe hiftory I now relate, is a well-informed men, and I found little difficulty in obtaining his fanction to a practice which was deemed conducive to the reftoration of of his child, and which might probably obviate the danger of contagion in a very numerous family.

" When I firft vifited my patient he was under the influence of the hot ftage, and there appeared not the fmalleft tendency to its folution by perfpiration. I therefore introduced into his chamber a free current of air. I then directed him to be placed in the middle of the floor, and the whole furface of his body to be fponged with cold vinegar.

" I faw the operation performed; and although he was at firft much averfe to it, yet he felt it fo cooling and refrefhing to him, that he never afterwards objected to a repetition of the experiment. The attendants, therefore, had recourfe to the application as often as he felt hot, or appeared to them to be fo, and with the moft beneficial effects. But they had the ftricteft injunctions, not to hazard the application, when he had the leaft chillnefs upon him, or when there was the fmalleft tendency to perfpiration. In either of thefe cafes, I conceive the practice to be highly dangerous; but during the hot ftage of fever, I have uniformly found, that the application of cold liquids reduces the frequency of the pulfe, and increafes the ftrength. It has alfo a wonderful effect in obviating the tendency to delirium, in diminifhing thirft, in increafing the efflorefcence of the fkin, and in inclining it to a gentle moifture.

" In the prefent cafe, thefe effects were ftrikingly exhibited indeed.

" From the apparent violence of the attack, and the early period at which the moft alarming fymptoms made their appearance, I had every reafon to prognofticate the approach of delirium, accompanied by thofe phenomena which precede the laft cataftrophe. On the morning, however, of the fourth day of fever, I found that he had had a good night, and was then very compofed. His tongue was moift; his pulfe reduced to 120 ftrokes in the minute; his fkin felt foft, and the febrile heat was much diminifhed.

" I fhould have obferved, that from the firft of my attendance, I had directed him to have a table fpoonful every hour, of equal parts of *aq. ammon. acetat. et aq. fontan.* And the attendants were inftructed to give him a fpoonful or two, occafionally, of the opening folution already mentioned, fo as to keep the bowels in a ftate of folubility. His common drink was cold water.

" This morning there appeared a little floughnefs on the tonfils; and I ordered him a gargle with *mel. rosa et hordeat.* acidulated ftrongly with the nitrous acid. The cold applications were conftantly ufed on the approach of the hot ftage; and to each dofe of his julep, a few grains of nitre were added.

" During the whole of this day, he continued to derive relief from this practice; and, on the morning of the 5th, his urine was nearly the colour of bright water. His tongue was clean and moift; his pulfe 105; the floughs in his throat had difappeared; and from this period he recovered rapidly.

" Seven of the family were fucceffively feized with the fame

 fpecies

ſpecies of fever; and, by a ſimilar treatment, were all ſpeedily reſtored to the moſt complete health.

"In one of the caſes, the moſt ſerious conſequences were manifeſtly combated ſucceſsfully by the practice deſcribed. In no inſtance was the fever protracted beyond the 5th or 6th day.

"I have thus ſketched the moſt prominent features of a ſolitary caſe of ſcarlatina, cured by the uſe of cold applications; and were it neceſſary to eſtabliſh the efficacy of the practice, I could adduce a very conſiderable number.

"During my attendance on this family, I caught the contagion. The ſymptoms of ſcarlatina were diſtinctly and rather ſtrongly marked; but by the timely application of a ſhower-bath, twice a day, the febrile affection was ſpeedily removed. I took no medicines.

"If the good effects of this practice be deducible from the cold produced by the proceſs of evaporation, are there not other applications which would more effectually and powerfully accompliſh the object propoſed?"

XIV. *Medical Cases*; by John Haxby, Phyſician, Pontefract.

3. A caſe of enlargement of one of the ſpinal vertebræ, gradually diſappearing on an enlargement of the trochanter major of the right thigh, which was ſucceeded by hydrocephalus, terminating fatally.

"W. S. aged nine years, had for ſome time laboured under immobility of the lower extremities, with conſtipation of the bowels, and a degree of dyſuria; owing to an enlargement of one of the ſpinal vertebræ.

"Cauſtics were applied to each ſide of the prominence, and kept open ſome time; during which there was a ſlight alleviation of his complaint. But as the amendment was not in proportion to his expectation, nor equivalent to the ſacrifice of his eaſe from the irritation of the cauſtics, about two months after their inſertion he allowed the ulcers to heal.

"Very ſoon after this, the trochanter major of the right thigh bone began to be enlarged; and in proportion as it increaſed in bulk, the diſeaſed ſpinal vertebræ was diminiſhed, till at laſt there was no inequality in the appearance of the ſpine.

"The trochanter continued to be enlarged for about a month; though great pains were taken to reduce the ſwelling by friction with ſpirituous embrocations, which was at length effected; and now he could walk with perfect eaſe, as far as could be expected from his reduced ſtrength; but he ſoon began to complain of pain in his head, which became more and more violent, ſo that when I ſaw him, (which was about a week after it had come on) he appeared to have every ſymptom of hydrocephalus internus, except ſtrabiſmus, which ſupervened in a day or two, when he died.

"Was the hydrocephalus, in this caſe, at all connected, as cauſe and effect, with the previous enlargement and ſubſequent diminution of the trochanter major and ſpinal vertebra?—Would the timely re-inſertion of the cauſtics have prevented hydrocephalus?"

This

This volume contains a variety of other important cases and hints, for which we must refer our readers to the work itself. As memoranda in the Materia Medica, we notice a case of tetanus * cured by the liberal use of wine; a case of epilepsy cured by musk and opium; cases of croup cured by hydrarg. muriat. mitis, and by the polygala seneca.

---

*A brief History of Epidemic and Pestilential Diseases, with the principal Phenomena of the Physical World, which precede and accompany them, and Observations deduced from the Facts stated.* By NOAH WEBSTER, Member of several American Societies, &c. 2 vols. 8vo. pp. 1301. Price 18s. in boards. London, Robinsons, &c.

THIS comprehensive and elaborate work commences with an account of the diversity of opinions respecting the cause and origin of pestilence. The author then presents his readers with historical views of pestilential epidemics, and the phenomena in the physical world which preceded, attended, or followed them, from the earliest accounts down to the year 1798. These subjects occupy the first volume.

The second volume begins with a tabular statement of the bills of mortality for the two last centuries, which the author introduces thus:

"Our accounts of diseases and the phenomena of the world, which appear to be connected with them, are altogether imperfect. But in the two last centuries we have a tolerable history of diseases, and occasionally an account of the seasons and remarkable occurrences. In the following tables the reader will find the bills of mortality for London, Augsburg, Dresden, Boston, one church in Philadelphia, with the bills of a few years for Paris and Dublin; to which are prefixed such of the remarkable phenomena of the elements as I have been able to collect.

"As winter makes a part of two years, the word *severe* is set against the year in which the winter began. Thus, against the year 1607, the word *severe* refers to the winter of 1607-8. The blanks denote, either that nothing singular occurred in those years, or that I have no account of the occurrences. Further enquiries might probably enable me to fill many of those blanks.

"Bills of mortality do not exhibit a complete view of epidemics, as some of the most remarkable, especially influenza, destroy but few lives; and the bills of the years when that disease alone prevailed, are remarkably low. It is often the immediate precursor, in spring, of pestilential diseases in autumn, in which cases the bills of the year are very high."

The second volume also contains remarks on the tables; on pestilential periods; influenza; on the order, connection, and progression of pestilential epidemics; on the extent of a pestilential state of air; conjectures on causes; means of prevention, &c.

---

* See Foreign Literature, p. 569.

*Considerations regarding Pulmonary Consumption.* By Thomas Sutton, M. D. Member of the Royal College of Phyficians, and Phyfician to the Forces; 8vo. pp. 120. London, Robinfons.

The leading objects of Dr. S. in this pamphlet appear to be to invalidate the common opinion refpecting the caufe of emaciation and death in phthifis. Obferving that many patients die of this difeafe, when attended with very little cough or expectoration, and certainly with no marks of purulent expectoration, he concludes that the fymptoms often depend on fome other caufe. In fuch cafes, he thinks the caufe of death "is fuch a decreafe of the ftimulating quality of the blood, as at laft to render it incapable of continuing the circulation. This inference is fupported by the appearance of the blood drawn, which contains a very fmall proportion of craffamentum."

At p. 19, Dr. S. gives the following opinion, viz. "It may not be improper in this place, to ftate an opinion regarding the fource of thofe happy feelings, high fpirits, and that conftant hope of a favourable termination of the difeafe, which have been obferved to attend confumptive patients.

"Pleafant and exhilirating fenfations are, I believe, common to all perfons under a certain ftate of debility from difeafe unconnected with uneafy feelings. They may be occafioned by the common ftimuli of intellectual exertion, of the circulation of the blood, of food, &c. upon the debilitated body; as it is evident, that the fame ftimulus has a greater effect upon perfons debilitated by difeafe, where no powerful agent continues to occafion further debility, than, cæteris paribus, upon thofe who are in health. I have experienced fuch fenfations after two fevere fits of typhus fever, when, being very debilitated, I had the fame pleafurable fenfations (Dr. Darwin's expreffion) as if I had been in cheerful company in health, and had drank moderately of wine. But ftriking inftances of fuch effects from debility are obferved by medical practitioners, in patients who have been tormented by inflammations in the bowels, which have ended in gangrene. In fuch cafes, it has often been obferved, that patients are in remarkably good fpirits, and cannot be readily convinced that they are in any danger. Thefe feelings are occafioned by debility, brought on by ficknefs, pain, want of fleep and of food, which is acted upon by the common ftimuli of intellectual exertion, of food, of the circulation of the blood, &c. and have the effect, in the way related, of producing a confiderable degree of cheerfulnefs, though unhappily of fhort duration. This cheerfulnefs and hope is more obfervable in phthifical patients, becaufe the difeafe is of confiderable duration, and becaufe the debility gradually increafes; and the patients, at leaft ten hours in the day, are, during the greater part of their difeafe, free from uneafy fenfations. But, while affected with pain, or tormented with the irritation of heat in the night, paroxyfms of hectic fever, there is no more cheerfulnefs and refignation in them, than in people labouring under equally unpleafant fenfations in other difeafes."

In Section IV. the author ftates his own caufe of phthifis, which is, is, *an obstruction in the mesenteric glands.* This hypothesis he supports by cases, and reasoning. We believe with Dr. S. that *tabes mesenterica* and phthisis are often combined; but we also believe that they may exist independently of each other, which he appears to doubt; for, at p. 31, he says: "Hence it appears to me, that phthisis pulmonalis is caused by a disease in the mesenteric glands, and that the tubercles in the lungs, and some other of its symptoms, are excited by sympathy."

Sect. VI. contains "General observations respecting the action of sympathy in consumption. VII. Predisposition. VIII. Cause of emaciation and debility, IX. On hectic fever;" the cause of which, Dr. S. believes to be, "an obstruction of the perspiratory organs arising from the defective circulation."

XIII. *On the* Cure. When Dr. Warren looked into any new medical work, which he had seldom leisure to do, he first examined the method of Cure; and if he found nothing new there, he searched no farther. Our author recommends the usual remedies, though he explains their operation somewhat differently from his predecessors. Emetics seem to be his favourites, as they were of Drs. Symonds and Reid.

The pamphlet is concluded by an Appendix, containing ten Cases, examined after death, which tend to confirm the author's opinions.

Though we observe several inaccuracies, and what we deem errors, in the above work, we are nevertheless convinced that it is well calculated to improve the theory and treatment of this important disease.

---

*A short Account of the Infectious Malignant Fever, as it appeared at Uxbridge, and its vicinity, in the Summer and Autumn of the year* 1799*; with a detail of the good effects of Yeast and Vital Air, in the different stages of that Disorder.* By a Medical Practitioner. 8vo. pp. 50. price 1s. 6d.

We are sorry that the ingenious author of this pamphlet has not given his name to it; for medical facts generally require all the aid they can derive from the authentication of a name.

The disease appears to have been the cynanche maligna in its worst form, and highly contagious among the poor. The symptoms agree with those described by other writers, but in the cure the author says:

"The manner in which I have usually administered the yeast, has been, by putting a tea spoonful, or more, according as it agreed with the stomach, into a quart bottle, and filling it up with mild porter: of this the patients took a glass full, every hour, or oftener, if they were thirsty.

"I have found it particularly useful in a great number of cases; and, therefore, I cannot avoid recommending it as a most powerful antiseptic, in malignant fevers. I have generally given it from the beginning, and persisted in its use, till a restoration to health took place.

"But

" But there is another remedy, not uſually recommended in this diſorder, that I have found particularly ſerviceable, *viz.* the oxygen gas, or vital air, inhaled into the lungs; it does not appear proper, at the beginning of the fever; but, when the ſymptoms of debility come on, and the eruption aſſumes a dark purple colour; then, I found a frequent exhibition of it to alter that appearance ſurpriſingly.

" When I firſt uſed the vital air, I prepared it from the black calx of manganeſe; but finding the proceſs took up more time than I could conveniently ſpare, and obſerving the difficulty there was, in inducing a patient to ſit up in bed, to inhale it, I made uſe of the following method, which is much more ſimple, and anſwers equally well, without occaſioning the patient any fatigue, or giving the attendants unneceſſary trouble.

" I firſt cauſed the doors and windows of the ſick perſon's chamber to be cloſed; and then, taking a chafing diſh with ſome live coals, throw into it half an ounce of purified nitre in powder, which immediately fills the room with a thick, white cloud, that continues wafting about for a conſiderable time.

" On examining a patient, during this operation, I never fail to find that it increaſes the pulſe; and, however low it may be, does, for a time, give it a degree of vigour and energy. In a few minutes more, the difficulty of breathing diminiſhes; the blood veſſels of the cheeks and lips become of a more florid hue; and a gentle perſpiration breaks out on the ſkin.

" This proceſs I direct to be frequently repeated, in the courſe of the day, and I have ſeldom ſeen it regularly perſevered in, without producing decided benefit."

---

*An Eſſay on the Analyſis of Mineral Waters.* By RICHARD KIRWAN, Eſq. F. R. S. &c. 8vo. pp. 279. London, Bremner. Price 7s.

SUCH is the well earned celebrity of Mr. K. as a chemiſt, that it is ſufficient merely to announce the ſubject and the author, in order to ſecure readers.

The term *mineral waters*, is ſpecially applied to ſuch waters only as are diſtinguiſhed by a peculiar colour, taſte, ſmell, or other obvious property, from common ſpring, lake, river, or other water, fitted for economical uſes. Mineral waters thus underſtood, have long attracted the attention of mankind by their *medicinal* powers. Theſe, indeed, can properly be inferred only from their repeatedly experienced effects; yet, even with *this* reſtriction, the knowledge of their contents muſt be deemed highly important, not only for the purpoſe of imitating ſuch as are found beneficial, in countries where Nature does not afford them, but alſo for the purpoſe of diſcovering the medical powers, and mode of action, of certain ingredients taken in a certain proportion, and a given degree of dilution, with a long train of conſequences that may in time be deduced from this knowledge. There are alſo many other points of view, in which an acquaintance with the contents of mineral neral waters muſt be deemed of importance; and we are convinced that the ſubject could not have fallen into more able hands.

The work is divided into Two Parts. In the firſt part is contained an account, 1. Of ſubſtances found in mineral waters. 2. Of the teſts of thoſe ſubſtances.

The ſecond part treats of the analyſis of mineral waters, viz. 1. Of the *common method*, by teſts, evaporations, cryſtallization, ſolution, precipitation, &c. to each of which Mr. K. ſtates his objections. 2. The new method, by determining the exiſtence and quantity of elaſtic fluids, by eſtimating the ſolid and liquid ingredients. 3. The uſe of ſpirits of wine in the analyſis of mineral waters. 4. Tables, 1, of the quantities of real acid in mineral acids; 2, of the quantities of acid abſorbed by different baſes; 3, of the quantity of each baſe abſorbed by each acid; 4, of the proportion of ingredients in neutral ſalts; 5, of the length in feet of a column of common air at different barometrical heights and different temperatures. 5. Appendix, containing new experiments on various ſaline ſolutions.

---

*Medical Facts and Obſervations*, Vol. 8, pp. 240. 4s. 6d. Callow.

The public are indebted for the preſent, as well as the former Volumes of this uſeful Publication to Dr. Simmons, a gentleman long eminent for his learning and abilities. As this collection of papers holds an equal rank with the preceding, for curioſity as well as intereſt, it will be in the hands of every perſon who is anxious for the improvement of medicine and ſurgery; little therefore need be ſaid in recommendation of it. This volume contains twenty-three papers, any of which might be ſelected for the entertainment of our readers; but we chooſe the following caſe, as it may tend to diffuſe the knowledge of a remedy, for a very diſtreſſing complaint, which may be eaſily obtained and adminiſtered by every one.

" Ann Fuller, a ſingle woman, aged forty-two years, has, at different times, in the courſe of the laſt five or ſix years, laboured under a ſuppreſſion of urine; and in ſome of thoſe attacks, no urine paſſed from the kidneys to the bladder for ten or twelve days each time; the catheter having been repeatedly introduced to determine this fact.

" In the years 1794 and 1795, ſhe was confined to her bed ſeven months in a ſtate of great agony. The pain extended acroſs the loins, and down the courſe of the urethra, and was frequently attended with violent and long continued vomiting of blood. In the courſe of this attack, the left ureter might be felt diſtinctly in the groin, enlarged to the ſize of an hen's egg, and extremely painful when preſſed. This was evidently occaſioned by the preſſure of calculi, which ſhe afterwards voided in great number, with blood in conſiderable quantities, frequently half a pint at a time, without any mixture of urine.

" For her relief a variety of remedies was had recourſe to, ſuch as as repeated bleeding and warm bathing, ſaline purgatives, emetics of different kinds, camphor and opium in large doſes, uva urſi, mephitic alkaline water, &c. To the camphor, combined with opium, which brought on a copious diaphoreſis, ſhe was more than once indebted for a mitigation of her painful ſymptoms. The mephitic alkaline water was tried repeatedly, in different forms, plain, and with additions, cold and warmed, but it conſtantly occaſioned pain of the ſtomach and vomiting.

" At length, the hæmaturia continuing, accompanied with a good deal of pain, and every remedy that had been adminiſtered having failed to relieve her effectually, Mr. Gabriel Allen, my aſſiſtant, ſuggeſted to me a trial of a decoction of peach leaves, from which he had occaſionally ſeen good effects in caſes of nephritis. He was firſt led, it ſeems, to the uſe of this remedy by a perſon, not of the medical profeſſion, who was much reſorted to by patients labouring under complaints of this kind, and who made a very ſuccefsful uſe in ſuch caſes, of an electuary, compoſed of honey, and peach leaves dried and powdered; together with a decoction or infuſion of the leaves.

" After having ſeen ſo many other remedies fail in this caſe, I was anxious to try the effect of this new medicine. I ſay *new*, for although different writers on the materia medica, mention the anthelmintic properties of the leaves, and likewiſe of the flowers, of the peach tree, I do not find that any of them have noticed their effects in affections of the urinary paſſages.

" A decoction was accordingly prepared, by boiling an ounce of dried leaves of the peach tree, (*Amygdalus Perſica*, Linn.) in a quart of water, till it was reduced to a pint and a half. Of the ſtrained liquor ſhe took a pint daily, and at the end of thirty hours after ſhe began the uſe of this remedy, ſhe voided clear natural urine, and in a few days recovered.

" From that time ſhe has conſtantly kept by her a quantity of the dried leaves, and on the leaſt return of the ſymptoms has had recourſe to the decoction again. Since that period, ſhe has had ſeveral ſlight returns of gravel, and has even paſſed ſome ſmall calculi, but ſhe has had no return of the hæmaturia. Her preſent comfortable ſtate of health ſhe attributes to the uſe of the decoction of peach leaves; at any rate, it ſeems to be deſerving of a trial in ſimilar complaints. I have tried it in a variety of inſtances beſides the one which is more particularly the ſubject of the preſent letter, and I am deceived if it is not a medicine of conſiderable efficacy in complaints of this kind. Upon theſe grounds it is that I have ventured to recommend it to your notice."

---

*Obſervations on the Effects of various Articles of the Materia Medica, in the Cure of Lues Venerea: illuſtrated with Caſes.* By JOHN PEARSON, ſenior Surgeon of the Lock Hoſpital and Aſylum, and the Public Diſpenſary; Reader on the Principles and Practice of Surgery, pp. 200, 4s. 6d. Callow.

**This work is extremely well calculated to calm the agitation in the** minds.

minds of thofe, whofe opinions have been unfettled on the treatment of the Venereal Difeafe, fince the introduction of the new remedies. The fituation the refpectable author has long held, gives him full claim to the confidence of the public; and the work evidently fhews, that it is the production of a mind well adapted for careful obfervation and found judgment. The author has given us a candid examination and eftimate of the confidence to be placed in the effects of the *lignum guaiaci, radix chinæ, radix farfaparilla, mezereum, cinchona, opium, cicuta, faffafrafs, juniperus, bardana, faponaria, dulcamara, juglans, lobelia fyphilitica, aftragalus exfcapus, ammonia præparata, terra ponderofa falita, certain preparations of mercury, mercurial fumigations, and vitriolic, marine, and nitrous acids.* In the chapter where the author inquires into the ill effects that fometimes attend the exhibition of mercury, we felect with pleafure, the following juft and manly remarks.

"Indeed, I am fo far from feeling alarmed or perplexed, at the examples of ill fuccefs which occafionally attend the exhibition of mercury, or from confidering thefe mifadventures as reflecting difparagement or difgrace on that mineral, that I am rather furprifed at the fuccefs which fo often attends the indifcriminate ufe of it.

"There is a defcription of men who fcatter abroad their crudities very liberally, in compendiums and effays; a clafs of productions, feldom calculated to convey information, but principally defigned to perform the office of a midwife, by bringing their compilers into public view. With the fpurious intelligence collected from thefe retailers of fcraps, many people furnifh themfelves with a ftock fufficient to undertake the cure of their own complaints; and, not uncommonly, impart the precious commodity to others who are lefs learned than themfelves.

"That mercury, conducted by men of fuch endowments, fhould often fail of doing good, nay, that it fhould frequently inflict great mifchief, would be according to the natural order of things: but, that it fhould ever prove finally beneficial, ought certainly to redound to the credit of a medicine, whofe falutary agency cannot be invariably fruftrated by all the blunders of hardy ignorance.

"He who fhall difcard all general rules, becaufe they admit exceptions, ought likewife, for the fake of confiftency, to renounce all fcience, becaufe human knowledge is fallible and imperfect.

"My opportunities of adminiftering mercury, have not extended to lefs than twenty thoufand cafes; and I feel myfelf fully authorifed to affert, that it is a remedy always to be confided in, under every form of lues venerea; and, where we have only that one difeafe to contend with, that it is a certain antidote, and as fafe in its operation as any other active medicine, drawn from the vegetable, or the mineral kingdom. Let me not be mifunderftood here, as if I meant to fay, that it is a certain and fafe remedy in the hands of any one who undertakes to difpenfe it. Quite the contrary:—for a multitude of indifputable proofs might be adduced, that ignorance and error often render it one of the moſt precarious and miſchievous medicines in uſe."

We cannot refrain quoting the following uſeful caution.

"Many perſons have taught, that during a courſe of mercurial inunction, it is unneceſſary to continue the friction until the ointment be abſorbed; and that the ſame medicinal effects will be obtained, by merely ſpreading it over the ſurface of the ſkin, as by the more laborious proceſs of rubbing it in as completely as poſſible. Nothing can be more at variance with truth than this inſtruction; nevertheleſs, a doctrine ſo peculiarly grateful to the feelings of indolent and irreſolute patients, has not failed to acquire conſiderable currency. I do, however, moſt ſtrenuouſly proteſt againſt this ſlovenly and inſufficient mode of applying the ointment; a mode which muſt finally end in the injury of the patient, and the diſgrace of the ſurgeon. But I do not think it ſufficient ſimply to oppoſe ſo deluſive and dangerous opinion, without urging it as a matter of no inconſiderable importance, that the patient himſelf ought always to perform the friction.

"There may be circumſtances indeed, under which an imperious neceſſity may conſtrain the violation of this precept; but, whenever it is infringed, it is always at the peril of the patient's ſafety, unleſs the aſſiſtant conduct it with an accuracy and dexterity which is ſeldom poſſeſſed by thoſe who undertake this diſguſting office. Many inſtances have fallen under my notice, where the ill ſucceſs of the ſurgeon could be manifeſtly traced to this ſource; and where a compliance with the advice I have now ſuggeſted, has been immediately attended with the deſired effect."

At the end of the work the author has drawn up ſome general concluſions, which are too valuable to withhold from our readers.

"1. The guaiacum, ſarſaparilla, mezereum, walnuts, opium, and Peruvian bark, have often removed ſome of the primary and ſecondary ſymptoms of lues venerea, and have alleviated others. They are likewiſe each of them capable of removing certain ſequelæ of lues venerea, where the farther adminiſtration of mercury would prove injurious. Yet, no ſatisfactory ſeries of evidence can be adduced, demonſtrating that any, or all of theſe vegetables, given ſingly, or combined, are competent to the eradicating of lues venerea from the animal body.

"2. It muſt be conceded, that certain indubitable ſymptoms of ſyphilis have diſappeared, during a courſe of the vegetable remedies; but the ſame ſymptoms have generally recurred, even at the very time when the patient was taking largely of the medicines which had produced this temporary benefit. Even where the patient has remained apparently well during five or ſix weeks, the diſeaſe has nevertheleſs always returned; and, what is worthy of particular attention, the ſame ſymptoms preciſely have recurred, which had been ſeemingly cured during the adminiſtration of the medicines alluded to. This fact may be conſidered as a proof, that venereal ſymptoms are not cured by them in any proper ſenſe; becauſe local appearances admit of a perfect cure by a mode of adminiſtering administering mercury, which ſhall nevertheleſs be inſufficient to ſecure the conſtitution.

" 3. The muriated barytes, and two of the mineral acids, when given to venereal patients, have the power of ſuſpending, for a limited time, the progreſs of the diſeaſe, and of removing many ſecondary ſymptoms; but they are not equal to the ſubduing of the virus, and freeing the conſtitution entirely from the effects of that deſtructive malady. They may likewiſe be employed with great advantage in thoſe phagedenic ulcers of the genitals, and of the groin, which may be claſſed among the ſequelæ of ſyphilis.

" 4. The nitric and nitrous acids have removed both the primary and ſecondary ſymptoms of ſyphilis; and, in ſome inſtances, it ſeems, that the former have not recurred, nor have ſecondary ſymptoms appeared, at the period they commonly ſhew themſelves, when the cure has been imperfect. But, as far as my own experience extends, and that of many reſpectable friends, who are connected with large hoſpitals, a permanent cure has never been accompliſhed by theſe acids, where ſecondary ſymptoms have been preſent.

" The ſame acids, when exhibited with the utmoſt care and attention to many patients labouring under the primary ſymptoms of the venereal diſeaſe, and where they have agreed perfectly well with the ſtomach, have been nevertheleſs, found inadequate to the cure of thoſe ſymptoms. Indeed, the failures which have occurred, both in my own practice and that of many of my ſurgical friends, have been ſo numerous, that I do not think it eligible to rely on the nitrous acid, in the treatment of any one form of the lues venerea.

" But, while I am obliged thus to detract from the ſuppoſed merits of the nitrous acid as an antidote againſt lues venerea, I would by no means wiſh to ſee it exploded as a medicine altogether uſeleſs in that diſeaſe.

" Where an impaired ſtate of the conſtitution renders the introduction of mercury into the animal ſyſtem inconvenient, or evidently improper, the nitrous acid will be found capable of reſtraining the progreſs of the diſeaſe, while, at the ſame time, it will improve the health and ſtrength of the patient. On ſome occaſions, this acid may be given in conjunction with a courſe of mercurial inunction; and it will be found to ſupport the tone of the ſtomach; to promote the appetite; to determine powerfully to the kidneys, and to counteract in no inconſiderable degree the effects of mercury on the mouth and fauces. Theſe advantages are by no means unimportant; and certainly entitle the gentlemen who have been active in promoting the introduction of this acid into general practice, to the gratitude of the public.

" I will not preſume, however, to aſſert, that we have yet learnt all that can be known, of the beſt mode of exhibiting this medicine; nor will I ſuppoſe that we have arrived at the *ne plus ultra* of its virtues. Yet, in the preſent ſtate of our information upon this ſubject, it would by no means be warrantable to ſubſtitute the nitrous acid in the place of mercury, for the cure of venereal complaints; nor to permit the knowledge we have gained reſpecting ſome uſeful properties of the former, to ſeduce us to reject what a long courſe of experience has taught us of the certain efficacy of the latter."

